# Management of hypersensitivity in a patient with late-onset Pompe disease experiencing recurrent infusion-related reactions

**DOI:** 10.1007/s00415-025-13357-w

**Published:** 2025-09-11

**Authors:** Daniel H. Mendelsohn, Natalia Garcia-Angarita, Andreas Hahn, Benedikt Schoser, Stephan Wenninger

**Affiliations:** 1 Friedrich-Baur Institute at the Department of Neurology, University Hospital, LMU Munich, 80336 Munich, Germany; 2 Department of Child Neurology, Justus-Liebig-University Giessen, 35392 Giessen, Germany

Dear Sirs,

Pompe disease (glycogen storage disease type II, GSD II) is a rare autosomal recessive lysosome storage disease (LSD) caused by a deficiency of the enzyme acid α-glucosidase (GAA), leading to the progressive accumulation of glycogen in lysosomes and cytoplasm. The main affected tissues are skeletal, cardiac, and smooth muscle [1]. Late-onset Pompe disease (LOPD) has a broad phenotypic spectrum. The age of onset ranges from early childhood to the sixth decade of life. Clinical presentations range from asymptomatic hyperCKemia to progressive proximal muscle weakness and diaphragmatic weakness with subsequent respiratory insufficiency [2]. Diagnostic hallmarks include initial screening via GAA activity testing in dried blood spots as well as confirmation via GAA gene mutation analysis, with biallelic pathogenic variants confirming the diagnosis [3].

The establishment of enzyme replacement therapy (ERT) with recombinant human GAA (rhGAA) has vastly improved the clinical outcome of LOPD via stabilization or slowing of disease progression [4]. Therapeutic options include first-generation alglucosidase alfa, as well as second-generation agents such as avalglucosidase alfa and cipaglucosidase alfa combined with miglustat, whose availability may vary depending on regional regulatory approvals. Pompe disease requires a high dose of therapeutic protein (20 mg/kg) compared to other LSDs as skeletal muscle uptake of enzyme is limited. Proposed mechanisms include nonproductive clearance of recombinant GAA by nonmuscle tissues and the inherently low mannose-6-phosphate content of the enzyme restricting receptor-mediated uptake, among other hypotheses such as low resting muscle blood flow or intracellular mistargeting [5]. Although high cumulative protein exposure during ERT is sometimes suggested as a factor contributing to antibody formation, this relationship is not straightforward. In fact, higher antigen doses may also induce tolerance. Conversely, protein misfolding or aggregation at high concentrations could expose novel epitopes and thereby promote immune recognition. Thus, the risk of ADA development is likely determined not by dose alone, but by a combination of patient-specific and product-related factors[6]. However, while high titers of IgG anti-rhGAA are associated with poor response to ERT in infantile-onset Pompe disease (IOPD) [7], the role of anti-rhGAA IgG in LOPD is less clear, as 90% of LOPD patients develop IgG ADA and no clinical correlation could be observed [8].

Infusion associated reactions (IARs) may arise even after several years of ERT without prior adverse events and may occur during or shortly after ERT administration and encompass a broad spectrum of immunological mechanisms, including IgE-mediated anaphylaxis [9]. The severity of IARs ranges from mild transient reactions to severe life-threatening consequences [10]. These reactions pose a substantial challenge, often necessitating therapy interruption or discontinuation. Interruption of ERT in LOPD leads to deterioration in clinical outcomes [11]. IARs can be managed through premedication, modified regimens, immunomodulation or desensitization protocols [12]. We present the case of a patient with LOPD and recurrent hypersensitivity reactions to ERT who was successfully desensitized via an individualized desensitization protocol.

A 46-year-old white male patient presented with an approximately 30-year history of progressive proximal tetraparesis, pronounced axial muscle weakness, bilateral Trendelenburg gait, and hyperlordosis accompanied by progressive back pain. The patient first experienced intermittent calf myalgia in early childhood. In his early twenties, lumbar pain and progressive hyperlordosis developed. By 35 years, generalized myalgia of the lower limbs (thighs and calves), reduced maximum walking distance, and abdominal muscle weakness with asymmetric bulging were noted. At 40, bilateral hip flexor weakness led to an inability to perform deep squats. By 44, rising from the floor required the Gowers’ maneuver. Pulmonary function tests showed a forced vital capacity (FVC) of 75% predicted and a maximal inspiratory pressure (MIP) of 60% predicted in the upright position. Serum creatine kinase (CK) activity was elevated at 411 U/L. Genetic testing at age 46 confirmed compound heterozygous mutations in the GAA gene (c.−32-13T > G and c.877G > A).

At age 46, ERT was initiated via biweekly avalglucosidase alfa infusions (20 mg/kg). Baseline neuromuscular assessment revealed a quick motor function test (QMFT) score of 47/64, a six-minute walk test (6MWT) distance of 507 m (70% predicted) and an FVC of 77% predicted in the supine position. The modified Rasch-built Pompe-specific activity (R-PAct) scale score was 20/34. After seven months of ERT, reassessment showed a decline in assessments with a QMFT score of 40/64, 6MWT distance of 420 m (58% predicted), and a FVC of 67% in the supine position. The modified R-PAct score was 17/34.

After nine months of home-based therapy, the patient developed urticarial reactions, which, according to published recommendations [10], led to a resumption of ERT at a Pompe expert center. Hospital-based infusions with premedication consisting of 100 mg methylprednisolone and 4 mg dimetindene were instituted but failed to prevent further reactions, including prolonged urticaria and chest tightness, consistent with a grade 3 IAR. Consequently, ERT was switched to alglucosidase alfa. However, immediate recurrence of IARs occurred after the first infusion. Therapy was paused for four weeks, and the patient was referred to the allergology department for intradermal testing, which revealed a positive immediate-type hypersensitivity reaction at a 1:10 dilution, indicating an IgE-mediated allergic reaction.

Following this first allergological consultation, extended premedication was initiated, consisting of 180 mg oral fexofenadine the evening before and on the morning and evening of the infusion day, along with 100 mg intravenous prednisolone and 4 mg intravenous dimetindene administered immediately prior to ERT. Despite this intensified regimen, urticaria and chest tightness occurred again at lowest infusion rate of 1mg/kg/h, prompting a second allergology consultation. After an 8-week pause, ERT was reinitiated at a reduced maximum infusion rate of 1.98 mg/kg/h under further escalated premedication: 180 mg fexofenadine (evening before and 1–0-1 on the day of infusion), 125 mg intravenous methylprednisolone, 4 mg intravenous dimetindene, and 300 mg subcutaneous omalizumab three days prior to the infusion. This protocol was well tolerated and subsequently maintained, with gradual increases in infusion rate over the following months without recurrence of infusion-associated reactions. In the absence of further infusion-related adverse reactions, the maximum infusion rate was gradually increased to 2.48 mg/kg/h after 6 weeks, 2.97 mg/kg/h after 10 weeks, and 3.47 mg/kg/h after 18 weeks. This cautious approach was essential to minimize the risk of renewed hypersensitivity reactions.

Immunologic monitoring included assessments of ADA and complement activation. Blood for ADA and complement activation monitoring was drawn prior to the respective infusion. After seven months of ERT, anti-avalglucosidase alfa (AVAL) IgG antibodies (1:1600) were detected and remained stable. Anti-AVAL IgE antibodies, measured after the IAR, showed a slight elevation to 0.35 kUA/L (quantitation limit: 0.35 kUA/L). Following the initial IAR under avalglucosidase alfa (AVAL), the patient received a single infusion of alglucosidase alfa (AL). Subsequently, anti-alglucosidase alfa (AL) IgG antibodies (1:400) were detected following the single AL infusion. Anti-AL IgE antibodies remained negative. Complement activation marker C3a was consistently negative.

Under the omalizumab-facilitated desensitization protocol, avalglucosidase alfa infusions were successfully reintroduced. Notably, after the fourth infusion following the reintroduction of avalglucosidase alfa, premedication with 4 mg dimetindene was accidentally omitted. Subsequently, the patient developed a minimal urticarial reaction localized to the elbow of the infusion arm. The reaction was promptly treated with 4 mg intravenous dimetindene and did not necessitate interruption of the infusion. The patient tolerated the reintroduced infusions well without recurrence of hypersensitivity symptoms. Muscle strength and respiratory function remained stable over the follow-up period of 8 months. The QMFT score was 43/64 and the 6-MWT distance was 449 m (62% predicted). Modified R-PAct score was 20/24. FVC in the supine position was 75%. Table 1 summarizes the treatment timeline, focusing on ERT regimens, IARs and corresponding interventions.

**Table 1:** ERT timeline with IARs and interventions

Week	ERT regimen	Max infusion rate	Reaction	Intervention
1	Initiation of ERT with Avalglucosidase	4.16 mg/kg/h	Well tolerated	None required
31	First IRA after home-infusion	4.16 mg/kg/h	Urticaria of arms, chest and abdomen	Treatment in emergency department via i.v. corticosteroids
45	Hospital-based infusion under premedication with 4 mg of dimetindene and 100 mg of prednisolone i.v	4.16 mg/kg/h	Urticaria of arms, chest and abdomen	Treatment with 4 mg dimetindene and 200 mg prednisolone i.v.; ERT paused for 4 weeks
50	Switch to alglucosidase under premedication with 4 mg of dimetindene and 100 mg of prednisolone i.v	4.16 mg/kg/h	Urticaria of arms, chest and abdomen	Infusion stopped due to IAR; emergency treatment with 4 mg dimetindene and 200 mg prednisolone i.v.; ERT paused for 8 weeks; Allergological consultation
59	Reinitiation of avalglucosidase under extended premedication: fexofenadine 180 mg evening before and 1–0-1 on infusion day; dimetindene 4 mg + prednisolone 100 mg i.v. preinfusion	4.16 mg/kg/h	Urticaria of arms and chest tightness	Infusion stopped due to IAR; emergency treatment with 4 mg dimetindene and 200 mg prednisolone i.v.; ERT paused for 8 weeks; Allergological consultation
67	Avalglucosidase infusion at reduced max. rate; premedication: fexofenadine 180 mg (evening before + 1–0-1), prednisolone 125 mg i.v., dimetindene 4 mg i.v., omalizumab 300 mg s.c. 3 days prior to infusion	1.98 mg/kg/h	Well tolerated	Maintenance of treatment protocol; conditional on absence of further IRAs
73	Accidental omission of dimetindene as premedication	1.98 mg/kg/h	Urticaria limited to infusion arm	Treatment with 4 mg dimetindene
79	Slight increase in max. infusion rate after 6 weeks without IARs	2.48 mg/kg/h	Well tolerated	None required
83	Slight increase in max. infusion rate after 10 weeks without IARs	2.97 mg/kg/h	Well tolerated	None required
91	Slight increase in max. infusion rate after 18 weeks without IARs	3.47 mg/kg/h	Well tolerated	None required

IARs during ERT are a rare but well-recognized complication in LOPD and represent a significant challenge to maintaining long-term therapy. Continuous ERT is critical to stabilizing disease progression and preserving motor and respiratory function in LOPD [11]. IARs may be IgE- or non-IgE-mediated and are likely influenced by the high cumulative protein exposure intrinsic to ERT in Pompe disease [12].

While standard premedication protocols—including corticosteroids and antihistamines—often mitigate mild to moderate reactions, refractory cases necessitate alternative strategies [12]. Switching to a different enzyme preparation may reduce the risk of antibody-mediated infusion reactions, but is not always successful, as anti-GAA antibodies generally cross-react between alglucosidase alfa and avalglucosidase alfa. This was evident in our patient, who experienced recurrent responses after a single alglucosidase alfa infusion [6].

Omalizumab, an anti-IgE monoclonal antibody, offers a rational therapeutic option by downregulating FcεRI expression on mast cells and basophils, thereby reducing their sensitivity to allergenic stimuli [13]. Although its use for ERT-related hypersensitivity remains off-label, omalizumab has been successfully employed in managing allergic reactions to other biological agents. In our case, combining omalizumab with a slow, stepwise infusion protocol allowed the safe reintroduction of avalglucosidase alfa without the recurrence of severe hypersensitivity symptoms.

Initially, no specific IgE antibodies against rhGAA were detected. However, during desensitization, low-level anti-avalglucosidase alfa IgE antibodies (0.35 kUA/L) became detectable, indicating sensitization over time. Despite the emergence of specific IgE antibodies, the desensitization protocol remained effective, suggesting that omalizumab + antihistaminic preinfusions may be beneficial even in the setting of evolving immunologic responses.

Notably, our protocol incorporated a significant initial reduction in the infusion rate, which was then gradually increased to minimize the activation of mast cells and basophils. This cautious escalation strategy, combined with omalizumab and conventional premedication, proved successful under conditions of detectable IgG ADA.

In LOPD, the clinical relevance of IgG ADA remains controversial. Unlike in IOPD, high IgG titers have not been consistently associated with loss of efficacy or increased risk of IARs. While anti-rhGAA antibodies frequently develop in LOPD, neutralizing effects appear to be rare and do not clearly impact functional outcomes. In our patient, IgG titers against avalglucosidase alfa and alglucosidase alfa were of similar magnitude, consistent with the expected cross-reactivity of anti-GAA antibodies, as both enzymes share the same catalytic backbone [8].

The positive skin test, clinical presentation, and detection of specific IgE suggested an evolving mast cell-driven hypersensitivity mechanism. Omalizumab, by modulating IgE-mediated pathways, appears to be a valuable adjunct for successful desensitization [13].

Although the previous reports have demonstrated either desensitization protocols without targeted immunomodulation [14], or the use of omalizumab in enzyme-related hypersensitivity without systematic desensitization [15], our case is—to our knowledge—the first to integrate both strategies within a structured, stepwise protocol guided by immunologic findings. This tailored approach, combining mechanistic understanding of IgE-mediated hypersensitivity with gradual reintroduction of ERT, enabled safe and durable continuation of treatment in a patient with repeated IARs to both alglucosidase alfa and avalglucosidase alfa.

The limitations of this report include its single-case nature and the short follow-up duration of eight months. Broader validation in larger cohorts is needed to establish standardized protocols and assess long-term safety and efficacy.

Nonetheless, this case report highlights the potential of omalizumab as an adjunct to desensitization protocols, enabling the continuation of life-prolonging therapy in patients with otherwise refractory hypersensitivity reactions.

A multimodal desensitization protocol—including reduced infusion rate, stepwise premedication with fexofenadine, dimetindene, and intravenous corticosteroids, as well as the addition of omalizumab—enabled the safe continuation of ERT in this patient with LOPD despite the previous recurrent infusion-associated reactions. Individualized approaches, informed by immunological assessment and gradual desensitization protocols, can enhance patient safety and improve treatment outcomes. Broader adoption of such strategies, supported by future research and clinical guidelines, may contribute to advancing standards of care in this rare disease population.

## Data Availability

Datasets are available on reasonable request from the corresponding author.
